# Time-dependent RNA transcriptional profiling of abomasal mucosa in cattle infected with *Ostertagia ostertagi*

**DOI:** 10.1038/s41597-025-04640-6

**Published:** 2025-02-21

**Authors:** Clarissa Boschiero, Ethiopia Beshah, Mariam Bakshi, Peter Thompson, Xiaoping Zhu, George E. Liu, Wenbin Tuo

**Affiliations:** 1 Animal Parasitic Diseases Laboratory, BARC, Agricultural Research Service, USDA, Beltsville, Maryland 20705 USA; 2 Department of Veterinary Medicine, University of Maryland, College Park, Maryland 20742 USA; 3 Animal Genomics and Improvement Laboratory, BARC, Agricultural Research Service, USDA, Beltsville, Maryland 20705 USA

**Keywords:** Gene expression, Infection

## Abstract

In this study, we infected Holstein calves with *Ostertagia ostertagi* stage 3 larvae (L3) and determined gene expression profiles of abomasal fundic and pyloric mucosa by RNA sequencing (RNA-seq) at 3–5, 7–9, 10, and 21 days post-infection (dpi), which represent late L3 and early L4 (between 3–5 dpi), mid to late L4 (between 7–9 and 10 dpi) and adult stages (21 dpi) of the parasitic stage. Bioinformatics analyses were performed to profile the transcriptomic changes over time as well as between cattle abomasal tissues. The results will help understand the gastric responses of the host, especially the immune responses, at different phases of early nematode infection. Such an undertaking is crucial for an in-depth comprehension of host responses, where immunologic reagents for cattle are highly limited. The RNA-seq datasets generated in this study provide a vital data resource, allowing for future comparative analyses to similar data resources or using more advanced analytical technologies in mechanisms of host-parasite interactions.

## Background & Summary

Gastrointestinal (GI) nematode infections are endemic to all regions of temperate climates in pastured cattle^1,2^. One of the most pathogenic nematodes, *Ostertagia ostertagi*, is an important cause of economic losses in the global cattle industry^1^. Due to the slow development of host immunity, grazing animals are commonly re-infected and suffer from clinical disease manifested by reduced weight gain in calves and decreased milk yield in adult cows^2,3^. As shown previously, *Ostertagia* infection can cause a wide range of changes in GI functions and tissue morphology^3^, including hyperplasia of the gastric mucosa and changes in GI mobility and secretions such as reduced acids and increased hormone release into the circulation. Mucosal hyperplasia can result in significant alterations influencing gastric epithelial cell differentiation and functions^3^. Infected mucosa has the typical signs of inflammation, such as increased redness and tissue thickness, bleeding, especially when the worms are at the adult stage, and formation of nodules, which may be associated with the presence of worms in the gastric glands.

Currently, control of the GI nematodes relies on anthelmintics and pasture management, yet anthelmintic drug resistance is rapidly developing^2^. Thus, there is a need to develop alternatives to chemical drugs in GI nematode control, including immunologic interventions such as vaccines. Parasitic worms are complex organisms, and understanding how the host may respond to the nematode infection is critical for the rational design of anti-nematode vaccines^2^. However, our knowledge of molecular mechanisms of the host-parasite interaction during the early stage of infection is limited^4^.

Gene transcription is one of the most critical initial steps through which the genotype is conveyed to the phenotype of the host responses. High-throughput RNA-seq using next-generation sequencing of RNA transcripts followed by *in silico* analysis has become the most popular method for transcriptome analysis. RNA-seq analyses of normal cattle tissue samples, such as Cattle Gene Atlas and CattleGTEx Projects, have provided a valuable global resource and framework for understanding normal animal physiology^5,6^.

Few studies have been used global transcriptional analysis to explore the gene expression profiling at different stages of nematode infection in cattle. One study analyzed the transcriptomic responses in the fundic abomasa of Angus cattle infected with gastrointestinal parasites, including *O. ostertagi* and *C. oncophora*^7^. Another study focused on the transcriptomic responses in intestinal samples of cattle infected with *C. oncophora*^8^.

**Fig. 1:**
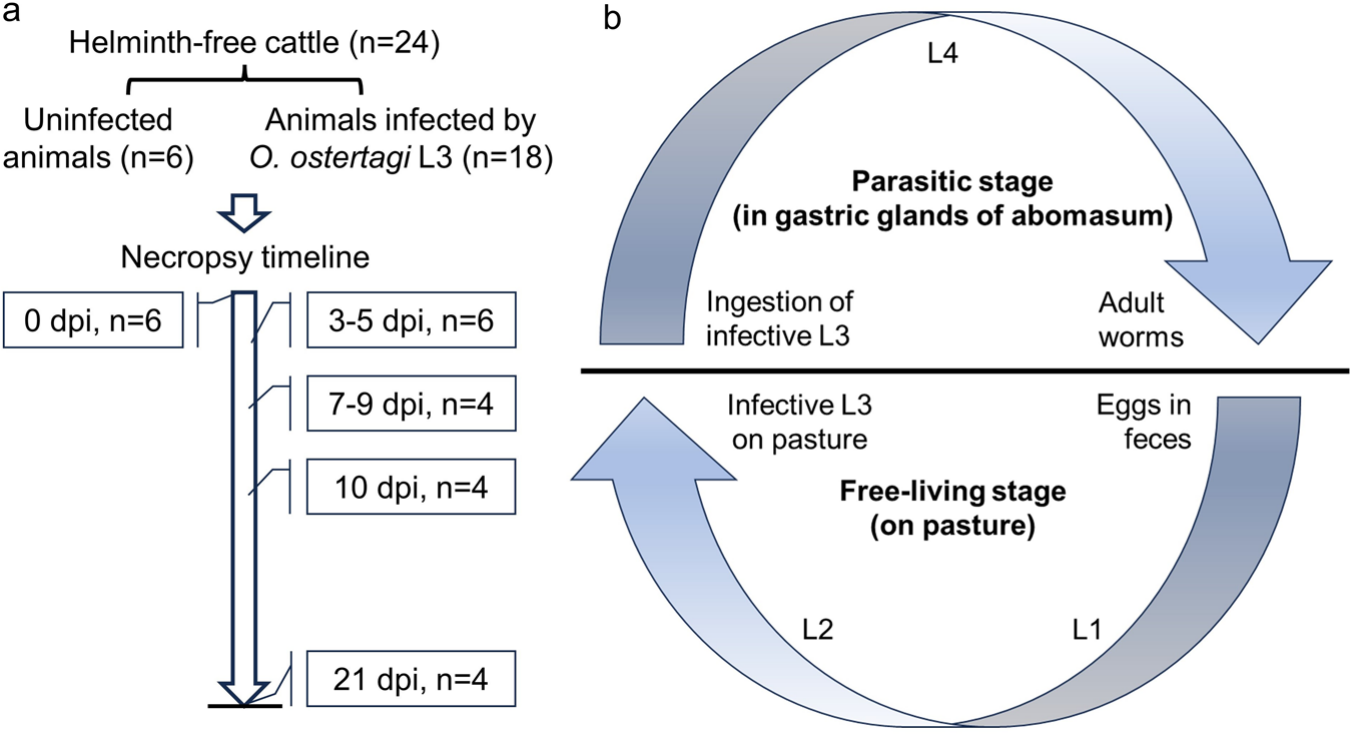
Schematic illustration of experimental design and *Ostertagia* life cycle. (**a**) Experimental design showing a timeline for tissue collection which correspond to late L3 to early L4 (3–5 dpi), mid to late L4 (7–9 to 10 dpi) and adult stages (21 dpi). A total of 24 Holstein steers were used in the experiment. Control animals (n = 6) were uninfected and received phosphate-buffered saline (PBS) only. Infected animals (n = 18) received oral infection by *O. ostertagi* stage 3 larvae on day 0 and were euthanized on Day 3, 5, 7, 9, 10, or 21 post-infection. During necropsy, abomasal fundic and pyloric mucosa were collected, snap-frozen in liquid nitrogen and stored at −80C untiled used. Frozen tissues were pulverized and stored at −80 °C until processed for total RNA isolation. (**b**) *Ostertagia ostertagi* life cycle. *O. ostertagi* has a direct life cycle which consists of free-living and parasitic stages. During the parasitic phase, animals are infected during grazing by ingesting infective L3 on the pastures. Following infection, the L3 quickly reach abomasum and burrow into the gastric glands where they develop into L4 and young adults. The adult worms enter into the abomasal lumen where they mate and produce eggs. Free-living stage starts when nematode eggs are released onto the pasture in feces. The eggs then hatch to L1 and molt into L2 and infective L3 on pasture. Next cycle begins when grazing animals ingest the infective L3.

In this study, we infected Holstein calves with *O. ostertagi* larval stage 3 (L3) and determined the transcriptomic profiles in the abomasal fundic and pyloric mucosa at 3–5, 7–9, 10, and 21 days post-infection (dpi), which represent late L3 to early L4 (between 3–5 dpi), mid to late L4 (between 7–9 and 10 dpi) and adult stages (21 dpi) of the parasite development^9^ (Fig. 1).

**Fig. 2:**
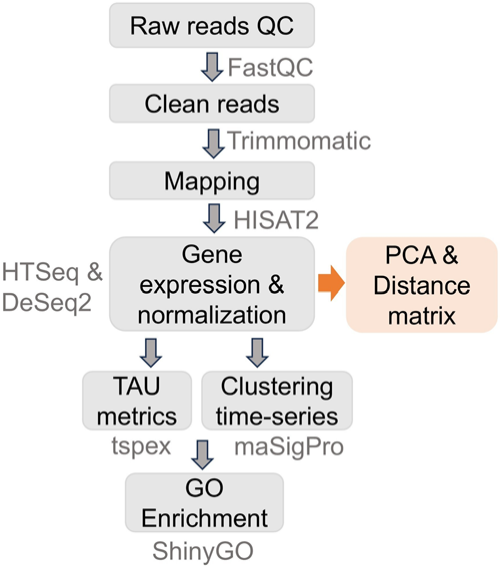
Bioinformatics pipeline flow chart for analysis of the cattle abomasal transcriptomes.

Bioinformatics analyses were performed to profile the transcriptomic changes over time as well as between abomasal tissues (Fig. 2). These comprehensive datasets document stage- and tissue-specific transcriptomic assessments of the cattle abomasa and can be a valuable resource for researchers aiming at infectious disease control and improvement of health, feed efficiency, production, and other economically important traits in cattle.

## Methods

### Animal infection and tissue collection

A total of 24 Holstein steers of 4–6 months of age were obtained from the Beltsville Agricultural Research Center (BARC) Dairy Unit, raised helminth-free from birth, and had free access to water and feed. All calves were weaned at two months of age. *O. ostertagi* propagation using helminth-free calves was conducted as described previously^10^. A total of 18 infected animals received a single oral dose with 200,000 *O. ostertagi* L3 per animal, and six control animals received PBS on day 0 (Fig. 1a). Animals were euthanized on day 0 (uninfected control; n = 6), day 3 (n = 3), day 5 (n = 3), day 7 (n = 2), day 9 (n = 2), day 10 (n = 4) or day 21 (n = 4) post-infection (dpi). Then, abomasal mucosa was collected at necropsy, snap-frozen in liquid nitrogen, and stored at −80 °C until used. Briefly, abomasum was removed from the abdominal cavity and abomasal content was drained through an incision along the lesser curvature. Abomasum was then cut open to expose the mucosal surface which was then briefly rinsed with cold PBS. Fundic mucosa was collected from the fundic region (pink in color) 1–2 inches from the omasum-abomasum junction, and pyloric mucosa was dissected from the pyloric region (pale in color) 1–2 inches from the pyloric sphincter. Since there were too few animals on 3, 5, 7, or 9 dpi, we combined the time points into 3–5 dpi or 7–9 dpi based on clustering patterns of principal components analysis^11^. Animal handling and infection by *O. ostertagi* was done according to the Animal use protocol approved by BARC IACUC (protocol number 19–012). Three samples (1 sample from fundic and 2 samples from pyloric tissues from 3 animals were eliminated from the study due to low data quality). Thus, the final datasets of this study were derived from a total of 24 animals with 23 fundic and 22 pyloric tissue samples.

### RNA isolation and RNA-seq library construction and sequencing

Frozen tissues were pulverized while submerged in liquid nitrogen using a Cryogenic Grinder (SPEX SAMPLEPREP, Metuchen, NJ, USA). Total RNA from each sample was extracted using TRIzol® (Thermo Fisher, Waltham, MA, USA). Purified RNA was resuspended in nuclease-free water and stored at −80 °C until use. RNA quality was assessed using Agilent Bioanalyzer 2100 (Santa Clara, CA, USA), and all samples with a RIN of 6 or greater were submitted for RNA sequencing to Novogene Inc. (Sacramento, CA, USA) and Azenta US Inc. (South Plainfield, NJ, USA) on an Illumina HiSeq 4000 (Illumina Inc., San Diego, California) sequencing machine.

### Pre-processing of sequencing data and alignment

All raw reads were quality-tested with FastQC v.0.11.9 (http://www.bioinformatics.babraham.ac.uk/projects/fastqc/). In addition, the quality of the reads was checked with the RseQC package (version 5.0.1) to obtain the read coverage over the gene body^12^. Clean reads were obtained by removing adaptors and low-quality reads with Trimmomatic (version 0.38)^13^ with the following parameters: TruSeq 3-PE.fa:2:30:10, LEADING:3, TRAILING:3, SLIDINGWINDOW:4:15, and MINLEN:36. The reads were mapped then to the cattle ARS-UCD1.3 reference genome^14^ using HISAT2 (version 2.2.1)^15^. BAM files were generated after the mapping and sorted using SAMtools (version 1.9)^16^. The mapping summary statistics of the 45 samples was deposited in figshare (10.6084/m9.figshare.25684446).

**Fig. 3:**
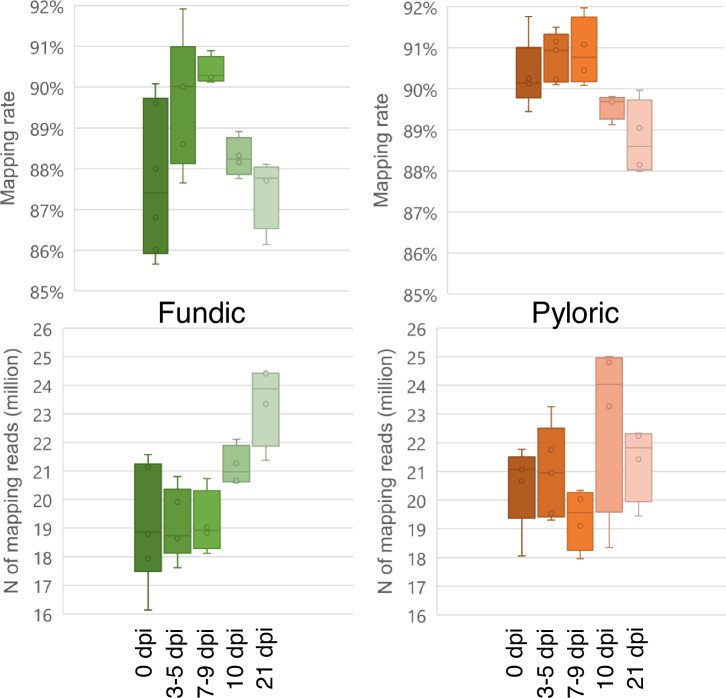
The mapping rate and numbers of mapped reads across different time points in cattle abomasal fundic and pyloric tissues.

The average input read count was 51.51 million per sample (ranging from 41.18 to 64.40 million). Following filtering, we obtained an average of 46.12 million clean reads, representing an average of 89.53% of reads that passed this filtering step, which was used for the subsequent analysis. The average percentage of uniquely mapped reads was 89.37% (85.66% to 91.97%) (Fig. 3). The reads mapped to unique genome locations and assigned to annotated regions of the cattle genome were used to calculate raw counts for each gene and downstream bioinformatics analyses.

### Gene expression and principal components analysis

HTSeq (version 2.0.2) was used to obtain the gene counts directly from the BAM alignment files using the HTSeq-count function^17^. We removed genes with zero counts and those located on mitochondrial and unplaced chromosomes in all samples. The normalized counts were obtained with DESeq 2 (version 1.38.3)^18^. DESeq 2 normalization utilizes the median of the ratios of observed counts to calculate size factors^19^.

Then, principal components analysis (PCA) and the distance matrix using the gplots package from R (version 4.2.1) were performed to assess sample quality and identify potential outliers and batch effects based on the normalized counts. The quality of the sequenced libraries was confirmed by examining the variance in gene expression among samples using PCA. The distance matrix was performed using the Euclidean method^20^.

### Time-specificity expression

The time-specificity indexes for each tissue were obtained based on the tissue-specific index Tau - τ^21^. This index ranges from 0, representing broad expression across all time points, to 1, representing specific expression at a particular time point. To obtain the τ index, the tissue-specificity calculator (tspex) tool was used (version 0.6.2)^22^. All expressed genes were used in this analysis, and each time point was compared to 0 dpi (control).

**Fig. 4:**
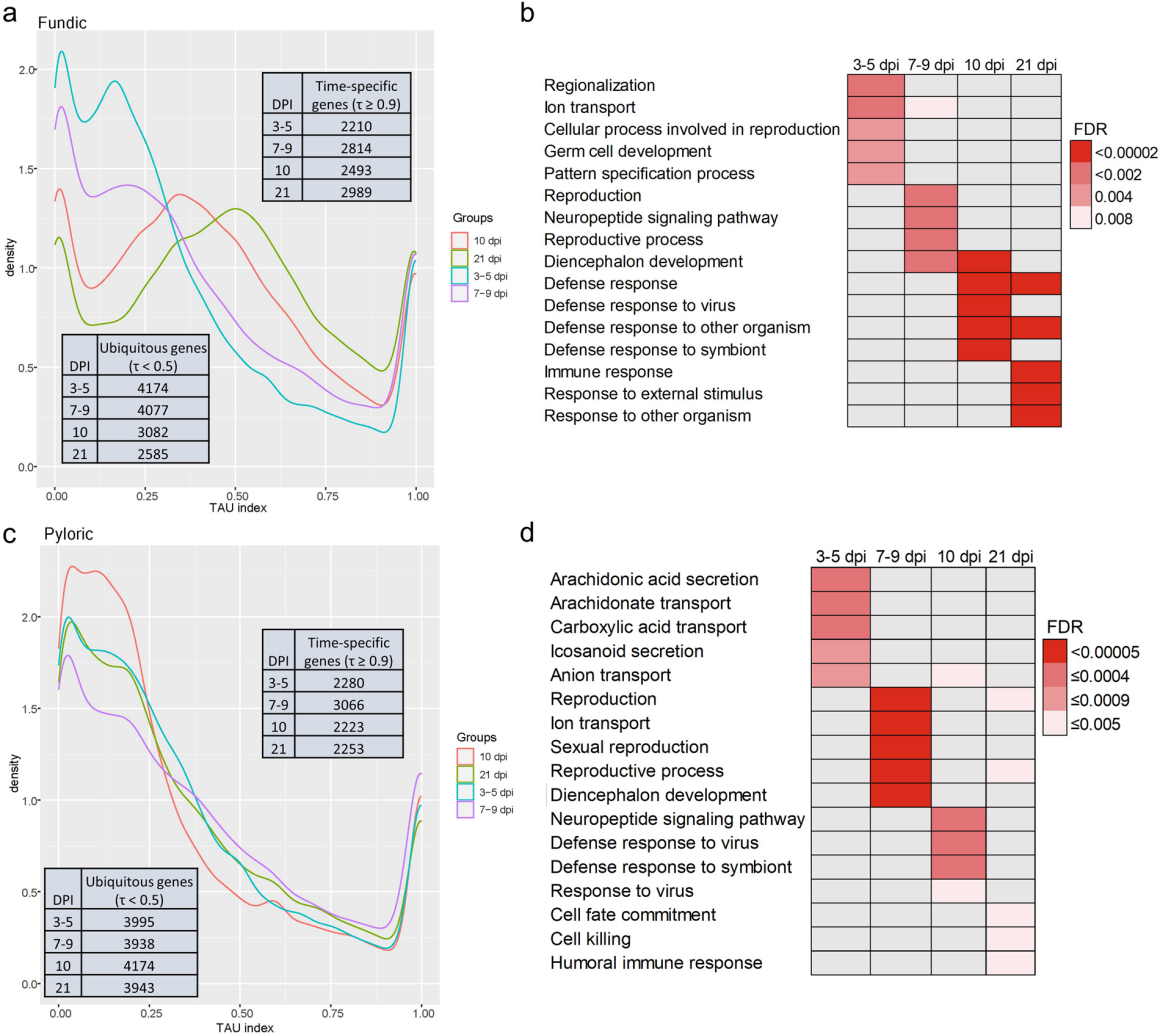
Time-specificity indexes (Tau index, τ) of two types of cattle tissues (fundic and pyloric mucosa) collected at four time points (3–5, 7–9, 10, and 21) post infection by *O. ostertagi*. compared to 0 dpi (control). (**a**) Time specificity for genes of the fundic tissue. (**b**) Top five GO enriched terms (FDR < 0.05) of the fundic time-specific genes. (**c**) Time specificity for genes of the pyloric tissue. (**d**) Top five GO enriched terms (FDR < 0.05) of the pyloric time-specific genes. The color gradient represents the FDR values of each enriched pathway.

For both tissues, time-nonspecific genes were higher than the time-specific genes at all time points (Fig. 4a,c). The time specificity gene list with Tau index in fundic and pyloric tissues considering four time points *vs*. control was deposited in figshare (10.6084/m9.figshare.25684455). It appears that, in the fundic mucosa, 10 and 21 dpi had fewer ubiquitous genes (τ < 0.5) than 3–5 and 7–9 dpi, while all-time points had a similar number of time-specific genes (τ ≥ 0.9) (Fig. 4a). In addition, in the fundic mucosa, there is a general trend of decreased non-time specific genes over time. Such a trend is not obvious in the pyloric mucosa (Fig. 4a,c). In the pyloric mucosa, the ubiquitous and time-specific genes are similar across the time points (Fig. 4c). The numbers of fundic time-specific genes are higher at 7–9 and 21 dpi. And the numbers of pyloric time-specific genes are similar at all time points except for those at 7–9 dpi, which are higher.

All time-specific genes (τ ≥ 0.9) in fundic and pyloric mucosa were further analyzed for GO enrichment (biological process or BP) using ShinyGO (version 0.77)^23^ with an FDR cutoff of < 0.05. The table with the enriched GO pathways for time-specific genes was deposited in figshare (10.6084/m9.figshare.25684452). These time-specific genes showed different biological functions relevant to the host responses to infections (Fig. 4b,d). There is a clear transition from non-immune responses to immune responses in the infected hosts over time. Time-specific genes at 10 and 21 dpi clearly revealed those with defense and immune response functions in both tissues. Some of the time-specific genes at 10 and 21 dpi in fundic and pyloric mucosa are related to host immune responses and defense pathways, including *CCL22*, *CCR2*, *GBP5*, *IFNE*, *IFNG*, *IL4, IL13*, *IL1A*, *IL1B*, *IRF4*, and *PRF1*. On the other hand, time-specific genes in early infection stages (3–5 and 7–9 dpi) in fundic and pyloric mucosa showed enriched pathways mainly related to ion transport, cell differentiation, and development.

### Time-course differential gene expression profile analysis

The time-course differential gene expression profiles for each tissue were obtained with maSigPro (version 1.72.0)^24^ to demonstrate specific clusters of gene expression between the different groups/time points from the normalized expression (Z-score of TPM). We defined four clusters (k) for each tissue with R^2^ > 70%. The list of expressed genes present in the four clusters for each cattle abomasal tissue with significant changes in expression over time was deposited in figshare (10.6084/m9.figshare.25684449). GO enrichment (FDR < 0.05) was obtained for each cluster using ShinyGO (version 0.77)^23^ to identify the biological functions during the different stages of the infection and deposited in figshare (10.6084/m9.figshare.25686084).

**Fig. 5:**
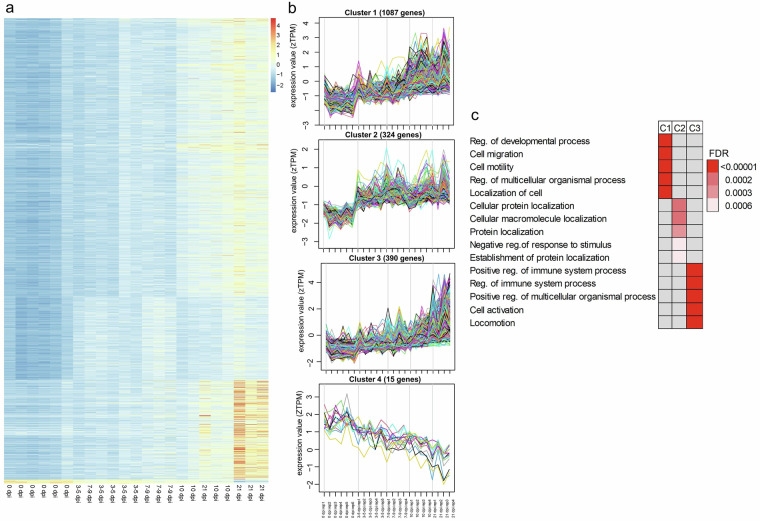
Gene expression profile clustering of all genes expressed by fundic tissue and identification of genes with significant changes in expression over time (0, 3–5, 7–9, 10, and 21 dpi). The time-course gene expression profiles are grouped into four clusters. (**a**) A heatmap of gene expression (rows) with significant changes in expression levels over time (columns). (**b**) Four clusters of expressed genes with significant changes in expression over time. (**c**) Top five GO enriched terms for clusters 1 (C1), 2 (C2), and 3 (C3) (too few genes in C4 for GO terms analysis). The color gradient represents the FDR values of each enriched pathway.

In the fundic tissue, 1816 genes were identified to show a significant expression across different time points (Fig. 5a). The heatmap shows non-overlapping, differential expression patterns in three clusters (Fig. 5a). Both fundic Clusters 1 and 3 have an increasing gene expression over time, although Cluster 3 genes show a higher expression towards 21 dpi than those of Cluster 1 (Fig. 5b). Cluster 4 demonstrates a higher expression at 0 dpi and a downward trend across the following time points. Genes in Cluster 1 were involved in several relevant biological processes, such as cell migration, adhesion, and motility (Fig. 5c). Genes in Cluster 2 were mainly related to protein localization and transport, membrane organization, and lymphocyte apoptotic process (Fig. 5c). In Cluster 3, genes were involved with several defense mechanisms such as immune response, T cell activation, leukocyte differentiation, regulation of immune system process, and cell migration and motility (Fig. 5c).

**Fig. 6:**
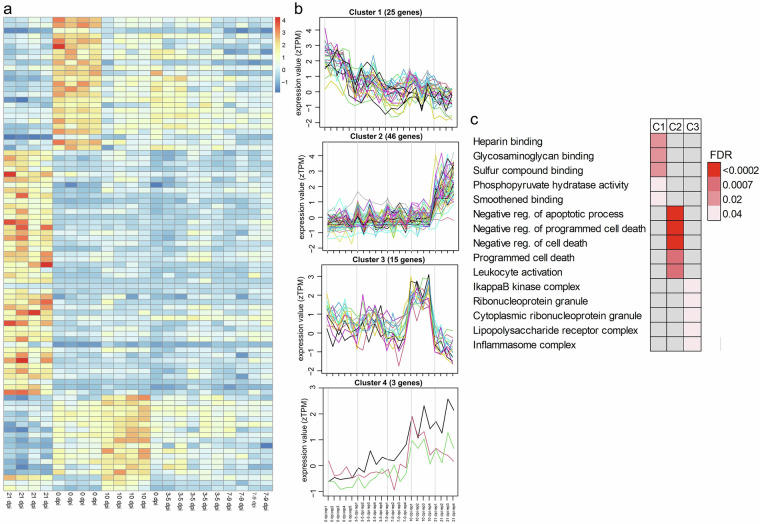
Gene expression profile clusters in pyloric tissue to identify genes with significant changes in expression over time (0, 3–5, 7–9, 10, and 21 dpi). The time-course gene expression profiles were grouped into four clusters. (**a**) Heatmap of the expression of the genes (rows) with significant changes in expression over time (columns). (**b**) Four clusters of expressed genes with significant changes in expression over time. (**c**) Top five GO enriched terms for clusters 1 (C1), 2 (C2), and 3 (C3). The color gradient represents the FDR values of each enriched pathway.

In the pyloric tissue, only 89 genes showed a significant expression at different time points (Fig. 6a), with decreasing expression over time for Cluster 1, but an increasing trend over time for Cluster 2 and Cluster 4 (Fig. 6b). Interestingly, gene expression in the Cluster 3 was higher at 10 dpi, but returned to basal levels at 21 dpi. In pyloric Clusters 2 and 3, relevant immune functions were also identified, such as increased cell and leukocyte activation (Cluster 2), regulation of immune effector process (Cluster 2), and elevated inflammasome complex (Cluster 3) (Fig. 6c).

## Data Records

The RNA-seq data were deposited in the NCBI Sequence Read Archive (SRA) under the BioProject number PRJNA994089 with accessions SRR25247730- SRR25247774^25–69^.

The mapping summary statistics of the 45 samples was deposited in figshare^70^. The time specificity gene list with Tau index in cattle abomasal fundic and pyloric tissues was deposited in figshare^71^. Moreover, the results of enriched GO pathways for time-specific genes in cattle fundic and pyloric tissues was deposited in figshare^72^. Furthermore, the list of expressed genes present in four clusters for each cattle abomasal tissue with significant changes in expression over time and their enriched GO pathways were deposited in figshare^73,74^.

## Technical Validation

### Quality control of RNA integrity

The quality of total RNA was examined by the Agilent Bioanalyzer 2100. All samples with an RNA integrity value of 6 or greater were subjected to sequencing.

### RNA-seq data quality

**Fig. 7:**
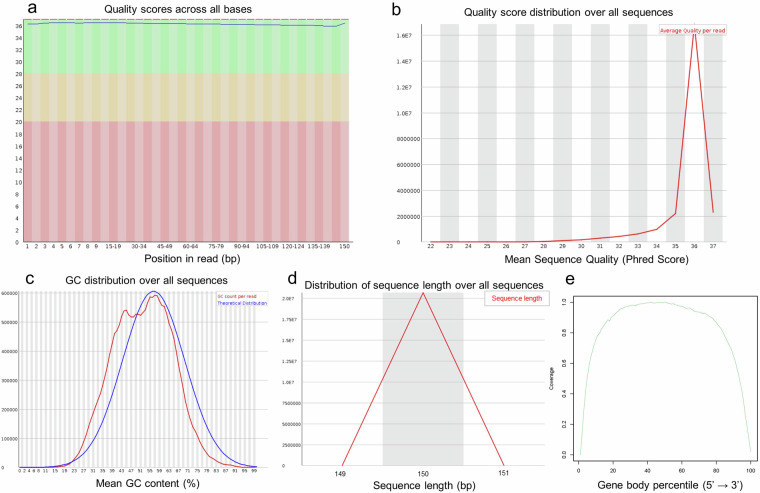
An example of RNA-seq quality check report from the FastQC tool from one representative cattle sample. (**a**) Representative quality score distribution for all 150 bp bases. (**b**) Representative quality score distribution of all sequences. (**c**) Representative distribution of GC content. (**d**) Representative distribution of sequence length. (**e**) Representative read coverage over gene body.

The quality of the raw reads from RNA-seq data was examined by FastQC. An example of a FastQC report for one of the 45 samples is shown in Fig. 7. In this example, the reads had high-quality values (Q > 30), as shown in Fig. 7a,b. The distribution of GC content was similar to the theoretical distribution with a normal distribution, indicating that the sample had no contamination (*i.e*., by adapters) (Fig. 7c). The sequence length distribution showed a peak at 150 bp, indicating consistency in fragment sizes (Fig. 7d). Then, the RseQC was used to assess the read coverage over the gene body, and no significant 5′ or 3′ end bias was identified (Fig. 7e). All of the 45 samples presented similar quality in the FastQC reports, implying high-quality sequences were used for the subsequent analysis.

**Fig. 8:**
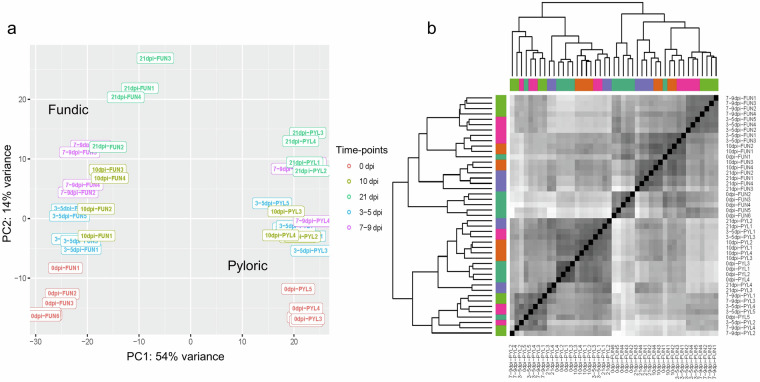
(**a**) Principal Component Analysis (PCA) plot of two abomasal tissues (fundic and pyloric) across five time points (0 dpi, 3–5 dpi, 7–9 dpi, 10 dpi, and 21 dpi). Time points are represented by different colors, as shown to the right. (**b**) Distance matrix of two abomasal tissues across five time points (0 dpi, 3–5 dpi, 7–9 dpi, 10 dpi, and 21 dpi). Different colors represent time points.

After the gene expression levels were obtained, we performed additional steps to assess the sample quality and identify potential batch effects and outliers. Analysis of all the expressed genes by PCA (Fig. 8a) and the distance matrix (Fig. 8b) showed that samples originating from the different tissues fell into two very distinct groups corresponding to fundic and pyloric mucosal tissues (PC1: 54%). The results also indicate that variabilities in gene expression explain tissue differences across time points (PC2: 14%). In general, the biological replicates for each time point are clustered together, with a better time point separation for the fundic than pyloric tissue (Fig. 8a). The distance matrix plot demonstrates that the fundic sample replicates clustered together, showing a high similarity, except 0 dpi replicate 1 (Fig. 8b). In the pyloric tissue, 10 dpi samples clustered together, showing high similarity, and the remaining samples showed different degrees of variability (Fig. 8b). Based on the PCA analysis, we combined 3 and 5 dpi animals as 3–5 dpi groups and 7 and 9 dpi animals as 7–9 dpi group, allowing for more replicates for each time point.

#### Ethics declarations

All animal procedures were conducted under the approval of the Beltsville Agricultural Research Center (BARC) Institutional Animal Care Protocol Number 19-012.

## Data Availability

In the current study, the following open-access software was used as described in the Methods section. For all the software, we used default parameters unless otherwise described, and no custom code was used. 1. FastQC (v. 0.11.9) was used to check the quality of raw FASTQ sequencing data: http://www.bioinformatics.babraham.ac.uk/projects/fastqc/. 2. RseQC (v. 5.0.1) was used to evaluate the quality of the reads and calculate the reads coverage over the gene body: https://github.com/MonashBioinformaticsPlatform/RSeQC. 3. Trimmomatic (v. 0.38) was used to trim adapters and filter quality reads: https://slequime.github.io/HTS-tutorial/trimming-trimmomatic.html. 4. HISAT2 (v. 2.2.1) was used to map sequence reads to the cattle bos taurus 9 genome: http://daehwankimlab.github.io/hisat2/. 5. SAMtools (v. 1.9) was utilized to sort and convert the SAM files to BAM format and index BAM files http://www.htslib.org/doc/samtools.html. 6. HTSeq (v. 2.0.2) was used to quantify gene expression by obtaining the gene counts from the BAM files https://github.com/htseq/htseq. 7. DESeq 2 (v. 1.38.3) was used to obtain the normalized counts: https://bioconductor.org/packages/release/bioc/html/DESeq2.html. 8. gplots (v. 3.1.3) was used to generate PCA and distance matrix plots: https://github.com/talgalili/gplots. 9. tspex (v. 0.6.2) was used to obtain the Tau index: https://github.com/apcamargo/tspex/. 10. ShinyGO (v. 0.77) was used to obtain the enriched GO terms: http://bioinformatics.sdstate.edu/go/. 11. maSigPro (v. 1.72.0) was used to obtain the time-course differential gene expression profiles: https://www.bioconductor.org/packages/release/bioc/html/maSigPro.html.
